# Occupational blood exposures in health care workers: incidence, characteristics, and transmission of bloodborne pathogens in South Korea

**DOI:** 10.1186/s12889-017-4844-0

**Published:** 2017-10-18

**Authors:** Ju Hyun Lee, Junhyeon Cho, Yung Jung Kim, Sang Hyuk Im, Eun Sun Jang, Jin-Wook Kim, Hong Bin Kim, Sook-Hyang Jeong

**Affiliations:** 1 Department of Internal Medicine, College of Medicine, Seoul National University, Seongnam, Republic of Korea; 2 Occupation Safety and Health Office, College of Medicine, Seoul National University, Seongnam, Republic of Korea; 3 Department of Internal Medicine, Seoul National University Bundang Hospital, 82 Gumiro 173, Bundang-gu, Gyeonggi-do 463-707 Republic of Korea

**Keywords:** Healthcare workers, Needlestick injury, Occupational blood exposure, Hepatitis C virus, Hepatitis B virus, Human immunodeficiency virus

## Abstract

**Background:**

Health care workers (HCWs) are at high risk for occupational blood exposures (OBEs) and transmission of bloodborne pathogens. This study elucidated the incidence rate and epidemiological characteristics of OBEs among HCWs and investigated the pathogen transmission rate for hepatitis B virus (HBV), hepatitis C virus (HCV), and human immunodeficiency virus (HIV).

**Methods:**

Self-reported OBEs from January 1, 2011 to December 31, 2015 were obtained from the electronic recording system. OBE incidence densities per 100 person-years and per 100 bed-years were calculated with a 5-year trend analysis. OBE characteristics and pathogen transmission rates were evaluated.

**Results:**

Among 10,452 HCWs and 1072 average yearly beds, 1076 OBEs were reported. OBE incidence rate was 5.6 cases per 100 person (full-time equivalent)-years and 20.3 per 100 bed-years. Incidence rate decreased and was significantly associated with a decrease of beds served per HCW. Housekeeping showed the highest OBE rate (14.8%) followed by doctors (8.5%) and nurses (6.2%). OBEs occurred in wards, emergency rooms, and operating rooms (38.1%, 13.3% and 12.2%, respectively) via percutaneous (86.7%) and mucocutaneous exposures (13.2%). Of OBEs associated with HBV (*n* = 133), HCV (*n* = 126), and HIV (*n* = 25), only one led to an infection (HCV; transmission rate of 0.8%). Neither HBV nor HIV infection occurred.

**Conclusions:**

OBE incidence rate in a Korean university hospital was 5.6 cases per 100 person-years and 20.3 per 100 bed-years and was related to HCW workload and work proficiency. Though the actual bloodborne pathogen transmission rate was low, efforts to prevent OBE should be made for hospital safety.

## Background

Healthcare worker (HCW) is defined as a person whose activities involve contact with patients or patient bodily fluids [1]. This includes nurses, physicians, pharmacists, technicians, morticians, dentists, students, contractors, attending clinicians, public safety workers, emergency response personnel, health care waste workers, first aid providers, and volunteers. An occupational blood exposure (OBE) is a percutaneous injury (e.g. a needlestick or cut with a previously used sharp medical device) or contact between a mucous membrane or non-intact skin (e.g. exposed skin that is chapped, abraded or afflicted with dermatitis) and blood, tissue, or other body fluids that may place a HCW at risk of hepatitis B virus (HBV), hepatitis C virus (HCV), or human immunodeficiency virus (HIV) infection [1].

The World Health Organization (WHO) estimated that each year, of the 35 million HCWs worldwide, 3 million experience percutaneous exposure to bloodborne pathogens (2 million to HBV, 0.9 million to HCV, and 170,000 to HIV [2]). These injuries result in 70,000 HBV infections, 15,000 HCV infections, and 500 HIV infections. Moreover, OBEs result in substantial psychological stress, such as job-related depression [3–5] and considerable management costs [6].

Though health care organizations maintain OBE control plans and government-regulated employee protection systems, healthcare-associated infections affecting HCWs remain poorly attended to, and very little systematic research regarding OBE and subsequent infections has been conducted. Moreover, the epidemiology and treatment of bloodborne infections, as well as hospital occupations, are dynamically changing. Thus, the continuous monitoring of OBE status and consequences is needed.

The aims of this study were to elucidate the incidence rate and epidemiological characteristics of OBEs among HCWs and to investigate the transmission rate of HBV, HCV and HIV through OBEs in a Korean university hospital.

## Method

### Study population

This retrospective cohort study involved HCWs at Seoul National University Bundang Hospital (Korea) over a 5-year period from January 1, 2011 to December 31, 2015. This resulted in 10,472 HCWs and 1072 average yearly beds, with a mean bed occupancy rate of 92%. Inclusion criteria for HCWs included full-time and part-time hospital staff for whom working times and occupation types were known for the full range of hospital employment. Full-time equivalent (FTE) work time was defined as 40 h a week and 52 weeks a year. In the case of interns and resident doctors on rotation duty, a monthly average number was calculated and converted to a 1 year equivalent. For part-time and temporary contract workers, dates and hours of employment were checked and converted to FTE units.

Several job categories, such as call center employees, public service workers, business logistics teams, funeral hall workers, and medical students without direct patient contact were excluded.

### Data collection

In 2011, this university-affiliated tertiary hospital integrated into the electronic health care recording system a user-friendly HCW self-reporting system for OBEs that was managed by the Occupational Safety and Health Office. In order to encourage self-reports, the hospital financially rewarded (~300 USD) departments that not only reported a high number of OBEs but also proposed reasonable ideas for OBE intervention and/or patient safety issues. This was done every 3 months and was started with the integration of the OBE reporting system (Oct 2010). This study was approved by the Institutional Review Board of Seoul National University Bundang Hospital (IRB No. B-1603/340–110), and informed consent was waived because the study used retrospective data.

Each OBE report included brief employee and exposure information. Employee information included the employee’s age, sex, designated department, occupation type, history of hepatitis B vaccination, and result of hepatitis B antibody test. Exposure information included exposure date, location, type (percutaneous injury, mucocutaneous exposure, and others), exposed body site, and route of exposure (e.g. preparing for procedure and sorting out materials, performing invasive procedures, organizing materials after procedures, handling clinical specimens, garbage disposal, and others). In the case of percutaneous injury, needle type and whether the needle was blood stained were checked. Mucocutaneous injury was defined as injury related to blood or bodily fluids. Several invasive procedures were specified, including blood sampling, operation, intravenous administration of medicine, blood sugar test, and needle insertion/removal. Post procedural material organization was subdivided into recapping, manual needle withdrawal from syringe, device or needle disposal, surgical drape organization, and tray transportation. Clinical specimen handling was subdivided into putting away clinical specimens, transporting clinical specimens, and others. Waste handling and disposal were subdivided into organizing a needle box, handling boxes containing infectious waste, cleaning the hospital, and others. If none of these options were applicable, one could add details in the report. HBV surface antigen (HBsAg), anti-HCV, and HIV test results of the source patients were determined when possible.

### Statistical analysis

OBE incidence rate was calculated as number of OBEs reported each year (over the course of 5 years) per 100 HCWs per year and per 100 average yearly bed number. The incidence rate was the number of new FTE cases per population at risk in a given time period, and we used the sum of the person-time of the risk populations as the denominator.

$$ \mathbf{Incidence}\mathbf{rate}=\frac{\boldsymbol{Numberof}\ \boldsymbol{OBE}}{\boldsymbol{Totaltimeexperienced}\ \boldsymbol{by}\ \boldsymbol{population}\ \boldsymbol{at}\ \boldsymbol{risk}}\times 100 $$

$$ \mathbf{Incidence}\mathbf{rate}=\frac{\boldsymbol{Numberof}\ \boldsymbol{OBE}}{\boldsymbol{Numberof}\boldsymbol{averageyearlybeds}}\times 100 $$

Annual trends in incidence rate were analyzed using Poisson regression analysis. Each item in the self-reporting system data was coded and entered into a database. SPSS v.22.0 software package for Microsoft Windows was used for analysis. A *P* value <0.05 was considered statistically significant.

## Results

### Incidence rate of occupational blood exposures during 2011–2015

**Fig. 1:**
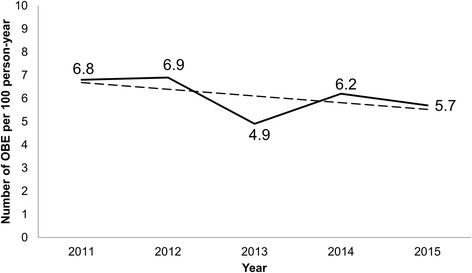
Incidence rate of occupational blood exposures at Seoul National University Bundang Hospital during 2011–2015. The solid line shows annual incidence rate. The dotted line shows the Poisson regression and incidence rate ratio, 0.94; 95% Confidence interval 0.90–0.98, *p* = 0.06

Over the 5-year study period, there were 10,452 HCWs, a 1072 average yearly bed number, and 1076 OBEs reported. The mean OBE incidence rate for 5 years was 5.6 per 100 person-years and 20.3 per 100 bed-years. Annual OBE incidence rate is illustrated in Fig. 1. The OBE incidence rate showed an annual 6% decrease, which was statistically significant (Incidence rate ratio [IRR], 0.94; 95% Confidence interval [CI] 0.90–0.98, *p* = 0.006). Incidence rate in 2013 was significantly lower than other years (IRR, 0.77; 95% CI 0.66–0.91, *p* = 0.002). In 2013, the number of employees sharply increased in preparation for the opening of a new hospital wing.

**Fig. 2:**
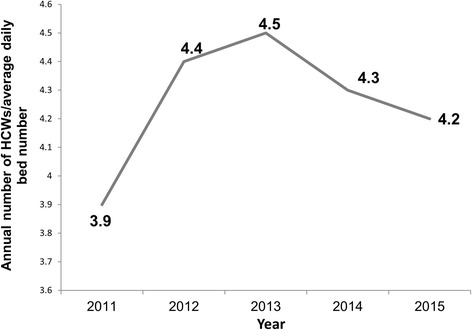
Annual HCW to average daily bed number ratio in Seoul National University Bundang Hospital in 2011–2015. The grey colored line shows annual HCW to average daily bed number ratio. It was 3.9 (3591/9110) in 2011, 4.4 (3972/908) in 2012, 4.5 (4841/1068) in 2013, 4.3 (5134/1189) in 2014, and 4.2 (5339/1283) in 2015, showing that the highest number was in 2013. HCW: healthcare worker

To determine whether OBE incidence rate was related to HCW workload, the annual number of employees per average daily beds served was calculated. It was 3.9 (3591/910) in 2011, 4.4 (3973/908) in 2012, 4.5 (4841/1068) in 2013, 4.3 (5134/1189) in 2014, and 4.2 (5339/1283) in 2015, showing that the highest number was in 2013 (Fig. 2).

### Epidemiological characteristics and related factors of OBEs

**Fig. 3:**
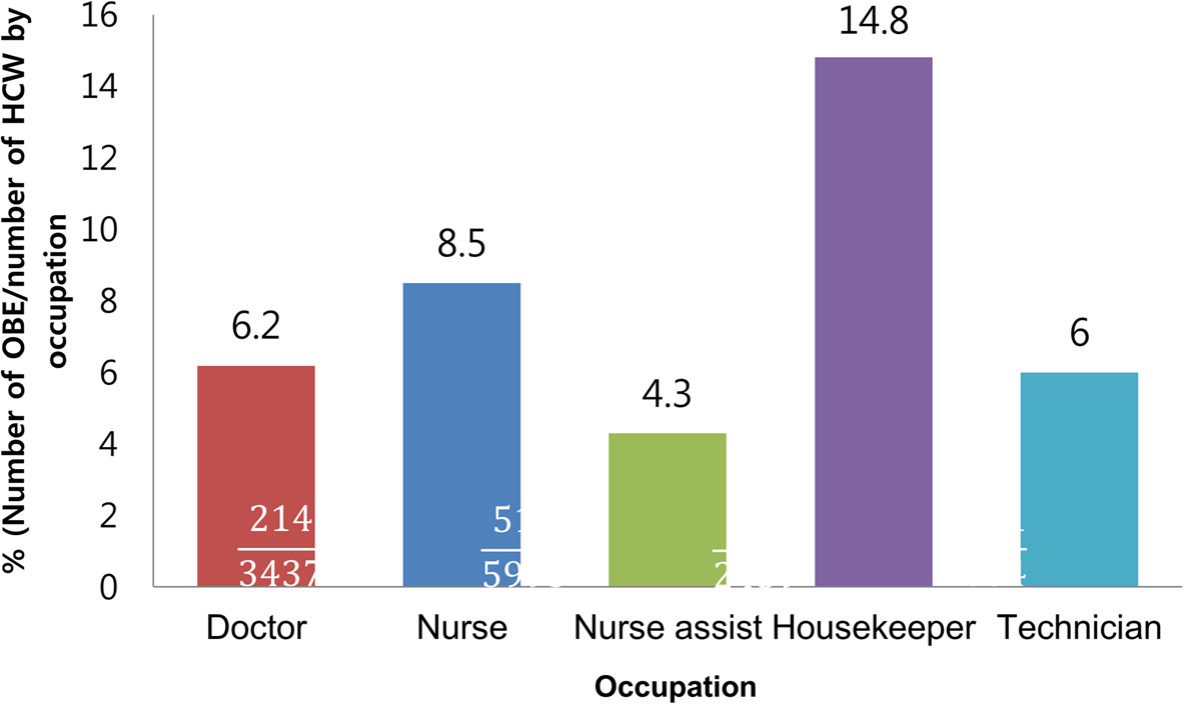
Exposure rate by occupation. Exposure rate was calculated by the number of OBE per number of HCW by occupation category. Housekeepers had the highest rate (14.8%, 131/884) and was followed by nurses (8.5%, 511/5995), doctors (6.2%, 214/3437), technicians (6%, 98/1609) and nurse-assists (4.3%, 118/2459). OBE: occupational blood exposure; HCW: healthcare worker

Detailed OBE characteristics are summarized in Table 1. Time between OBE occurrence to reporting was 0.5 ± 4.5 days (mean ± standard deviation [SD]), and 75% (802 cases) of all incidents were reported on the same day. The majority of incidents occurred in clinical wards (410 cases, 38.1%), followed by the emergency room (143 cases, 13.3%) and operating room (131 cases, 12.2%). According to occupational category, 511 (45%) of the OBEs occurred in the nurse group, 214 (20%) in the doctor group, and 131 (12%) in the housekeeper group. In the doctor group, interns (*n* = 76), residents (*n* = 54), and fellows (*n* = 33) accounted for most of the exposures (76%). Notably, the highest exposure rate (incidence of OBEs per 100 persons for each occupation) was found in housekeepers (14.8%, 131/884) followed by nurses (8.5%, 511/5995), doctors (6.2%, 214/3437), technicians (6%, 98/1609) and nurse-assists (4.3%, 118/2459) (Fig. 3). Circumstances leading to OBEs of HCWs are shown in Table 2. OBEs most often occurred while examining patients (45%, 482 cases) and was followed by organizing materials after procedures (28%, 298 cases), and disposing of garbage and others (17%, 175 cases,).

**Table 1:** Epidemiological characteristics and type of occupational blood exposures in healthcare workers during 2011–2015 (*n* = 1076)

Variables	Number (%)
Age^a^	34.6 ± 11.5
Sex (*n* = 1035)^b^	
Female/Male	851/184 (82.2/17.8)
Occupation	
Doctor	214 (19.9)
Nurses	511 (47.4)
Nurse-assist	118 (11.0)
Housekeeper	131 (12.2)
Technician	98 (8.9)
Others	4 (0.7)
Exposed place	
Clinical ward	410 (38.1)
Emergency room	143 (13.3)
Operating room	131 (12.2)
Laboratory	104 (9.7)
Treatment room	96 (8.9)
Outpatient department	84 (7.8)
Others	108 (10.0)
Working shift^c^	
Morning	515 (47.7)
Evening	438 (40.8)
Night	123 (11.5)
Exposed body site	
Hands and fingers	917 (85.2)
Eye, mouth, damaged skin	114 (10.6)
Others	45 (4.2)
Type of injury	
Percutaneous injury	933 (86.7)
Mucocutaneous exposure	143 (13.2)
Percutaneous injury (*n* = 933)	
Contaminated with blood	
Yes	670 (71.8)
No	263 (28.2)
Needle type	
Hollow bore needle	706 (75.7)
Variables	Number (%)
Solid needle	119 (12.8)
Others^d^	108 (11.6)

^a^Value represented mean ± standard deviation

^b^The cases with missing information were excluded

^c^Morning shift; 6 am-2 pm, evening shift; 2 pm–10 pm, night shift; 10 pm-6 am

^d^Lancet, scalpel, razor, scissors, trocar, staples etc.

**Table 2:** Circumstances leading to occupational blood exposures of healthcare workers during 2011–2015 (*n* = 1076)

Variables	Number (%)
Direct patient care	482 (44.8)
Invasive procedures	115 (23.9)
Blood sampling	111 (23.0)
Operation	94 (19.5)
Intravenous administration of medicine	67 (13.9)
Blood sugar test	40 (8.3)
Inserting or removing needle	31 (6.4)
Others	24 (5.0)
Organizing materials after procedure	298 (27.7)
Disposal of device or needle	148 (49.7)
Manual withdrawing of a needle from syringe	52 (17.4)
Recapping a used needle	42 (14.1)
Transportation of devices after procedure	33 (11.1)
Others	23 (7.7)
Disposing garbage and others	175 (16.3)
Organizing medical waste box	89 (50.9)
Cleaning the hospital beds or instruments	36 (20.6)
Organizing needle box	24 (13.7)
Organizing non-medical waste box	17 (9.7)
Others	9 (5.1)
Handling clinical specimens	76 (7.1)
Putting the specimen into the specimen bottle	65 (85.5)
Transporting the specimens	10 (13.2)
Others	1 (1.3)
Preparing procedure or sorting out materials	32 (3.0)
Others	13 (1.2)

**Fig. 4:**
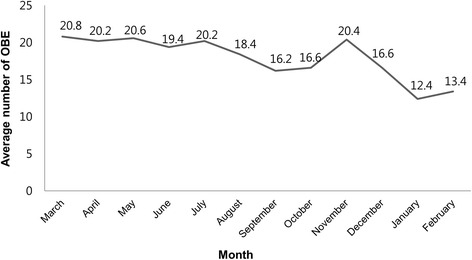
The average number of occupational blood exposure for each month of the year. The monthly average incidence of occupational blood exposures during the 5 years of study period was highest in March (20.8 cases) and April (20.2 cases), and then it decreased over the course of the year with the exception of November. January and February showed the lowest incidence throughout the year

The monthly OBE occurrence distribution is illustrated in Fig. 4. The monthly average OBE incidence was highest in March (20.8 cases) and April (20.2 cases) and then decreased over the year with the exception of November. January and February showed the lowest OBE incidence. New hospital employees enter in March, and November is the month of duty shift for Interns and Resident doctors in this hospital. Therefore, work proficiency may be related to OBE occurrence.

### Transmission rates of HBV, HCV and HIV

Of the reported 1076 blood exposure cases, the number of cases with available patient information was 681 for HBV, 680 for HCV, and 657 for HIV. Of these, the number of patients positive for HBsAg, anti-HCV, and anti-HIV were 133 (19%, 133/681), 126 (18.5%, 126/680), and 25 (3.8%, 25/657), respectively. Because of our hospital’s strict OBE system, all reported cases underwent initial blood tests within 24 h of the exposure, and almost all of them were followed for up to 6 months.

Only one HCW became infected with HCV, demonstrating a HCV transmission rate of 0.8% (1/126). Neither HBV nor HIV infection occurred. However, 12 HCWs received HBV vaccination and/or HB immunoglobulin, and 16 HCWs received prophylactic anti-HIV medications for 4 weeks.

The employee infected with HCV was an orthopedic surgeon fellow. During an operation, a scalpel contaminated with blood from a patient co-infected with HBV and HCV was dropped, cutting the surgeon’s heel. He presented with symptomatic, acute hepatitis C 8 weeks after the incident, undertook pegylated interferon and ribavirin therapy for 6 months, and recovered from HCV infection. In reaction to the incident, he experienced severe stress and transient depression.

## Discussion

This study found an OBE incidence rate of 5.6 per 100 person-years and 20.3 per 100 bed-years in a Korean university hospital. Among job categories, exposure rate was highest among housekeepers, followed by nurses and doctors. Of the 1076 incidents, one HCV infection developed, showing that the actual infection rate was low, despite the high exposure number.

Our five-year OBE incidence rates (5.6 per 100 person-year and 20.3 per 100 bed-year) were higher than those previously reported in South Korea (2.92–4.29 per 100 person year) [7–9]. A 2011 US study reported an OBE incidence rate of 2.67 per 100 person years. In 2014, the Exposure Prevention Information Network (EPINet) reported 24.7 OBEs per 100 average daily census of occupied hospital beds. It was calculated as the total number of needlestick injuries reported during a specific date range (numerator) over the number of occupied hospital beds in an institution for the same date range (denominator) [10].. In 2005, WHO reported the annual incidences of sharps injuries for 14 subregions. Incidence ranged from 0.18 to 4.68 sharps injuries per HCW per year [3]. Notably, existing OBE reports have shown variability in study durations, hospital characteristics, and methodologies for incidence rate estimation. They also share limitations related to insufficient study duration, lack of inclusion of every HCW and consideration of unreported cases [11–14].

In contrast, our study showed a relatively higher level of incidence rate compared to previous studies. The ease of OBE reporting in addition to the financial incentive provided by the hospital to departments with high OBE report rates and submission of HCW and patient safety ideas may have contributed to this finding. The high rate of same-day incident reports may support this notion. Studies show HCW may become desensitized to the dangers of blood exposure due to repetitive experiences of such incidents [15]. In this regard, emphasis on reporting OBE may continuously raise awareness of incidents and allow individuals to properly deal with OBE risks. To prevent OBEs and to encourage OBE reporting, the hospital has implemented orientation training for new employees and annual continuing education for all employees. Also, the hospital’s Occupational Safety and Health Office offers additional education for its high OBE risk departments.

Among the observed 5 years, OBE incidence rate was the lowest in 2013. In 2013, the number of caregivers increased sharply due to the opening of a new hospital wing. Nonetheless, blood sampling and other medical sharps work practices remained similar between the old and new hospital areas. The number of employees per bed was a record high in 2013 and was presumed to have had some impact on lowering the OBE incidence rate. Prakash K et al. reported that employee work intensity affected OBE incidence [16]. Ayas NT et al. identified that percutaneous injuries were more frequent in those with extended work hours [17]. Thus, the decrease in OBE incidence and corresponding increase in employee per bed in 2013 support excessive workloads, inadequate HCW to patient ratio, and fatigue as possible contributing factors to increased OBE.

Most OBE studies have included only nurses or have reported that nurses most often experience blood exposures, even when the entire medical team is included in the study. This is because the proportion of nurses is high among medical professionals. Our data indicated that nurses represented most of the reported incidents, in terms of absolute numbers, while the rate of occurrence was highest among housekeepers. The housekeeping group is involved in cleaning the hospital, disposing hospital and biomedical waste, organizing materials after procedures, and more. The current study found that blood exposures related to housekeeping work made up 19% of total incidents. Some were caused by discarding sharp devices in inappropriate places such as tables, trays, general wastebaskets, laundry, and more. As information on exposed blood cannot be traced back in these cases, more attention should be paid to post-exposure prophylaxis or infection. Saini S et al., showed that sanitary staff had a low level of knowledge about biomedical waste management rules (14%) when compared to doctors (80%) and nurses (60%) [18]. Mathur V et al. reported that regarding practices related to biomedical waste management, sanitary staff were ignorant on all the counts [19]. Megha K et al. identified that 48.7% sanitary staff were not trained [20]. Most hospital cleaning staffs are either part-time or temporary contract workers. Consequently, compared to other HCWs, they are less reachable by the hospital infection control management system. Future directions must focus on strengthening OBE prevention among the cleaning staff through safety education, safe disposal of sharp medical devices, as well as provision and use of protective equipment.

The monthly incidence of OBEs during the 5 years was the highest in March (20.8 cases) and April (20.2 cases) and decreased over the course of the year with the exception of November. In Korea, the new semester begins in March. Many new employees are hired during this time and are not accustomed to their work in March and April. Every November, 4th year resident doctors leave the hospital to prepare for a national examination, and little to no extra staff is hired to cover their absence. Our study showed that during the month of November, the overall work load increased and work proficiency transiently decreased. Therefore, employee job proficiency was associated with OBE incidence in this study. Likewise, Lee JJ et al. reported that needlestick injury events tend to occur in interns with lesser clinical skill and occur during a period when new interns and residents join the clinic [21]. Other studies have identified similar findings [22, 23].

Of the 1076 incidents, one HCV case led to infection with a 0.8% transmission rate. However, there were no HBV and HIV infections (0% transmission rate). Compared to exposure frequency, the rate of actual infection was low. As recommended by the Centers for Disease Control and Prevention, all HCWs at our institution are required to be vaccinated against HBV [24]. Accordingly, a majority (914) of the OBE reporting HCWs were anti-HBs positive. Previous studies have reported the risk of HBV transmission as 30% in susceptible HCWs without post-exposure prophylaxis or adequate hepatitis B vaccination; HCV infection risk as 0.5% (but considered null if exposure was to non-viremic patients); and <0.3% for HIV [25–29]. Eskandarani HA et al. reported no HIV, HBV or HCV transmission for over 10 years among OBE reporting HCWs in Denmark, showing that despite frequent OBE among HCWs, the risk of infection was low [30]. HCWs have relatively high anti-HBs positive rates due to mandated hepatitis B vaccinations. Moreover, patients who receive hospital treatment have low viral loads due to antiviral medication; therefore the frequency of actual infections is expected to be low. The present study showed a low seropositive conversion rate in HCWs following OBE. The hospital’s infection control management system (e.g. post-exposure prophylaxis administration) likely contributed to the low rate. However, even with low seropositive conversion rates, primary OBE prevention, in particular adherence to the universal precautions, must remain a priority at any healthcare setting worldwide.

In this study, the HCV infected HCW experienced depressive moods, anxiety, and weight loss caused by excessive stress. In addition to initial concerns, there may be extended periods of anxiety and distress as testing for bloodborne pathogens can last for months. In this regard, Sohn J-W et al. reported that HCWs with OBEs exhibited higher levels of anxiety and depression after injury [3–5]. Particular attention must be directed towards the psychological sequaeles of OBEs in HCWs.

This study had several limitations. First, the authors of this article acknowledge that sharps with injury prevention features (SIPFs) are an important intervention to prevent OBEs. There was some use of SIPFs in the hospital, however, the related data was unavailable, and therefore, our analysis did not address this. Second, the study was limited to HCW in a tertiary hospital; thus the results may not be generalizable to other settings. Nonetheless, this study examined a large and diverse population, and OBEs were studied with consideration to specialty and occupation. Third, our analysis does not take into account several occupational categories including medical students. Fourth, like all survey studies, response rate can be underestimated, and reporting bias is possible.

## Conclusion

Our study suggests that the incidence rate of OBE is higher than expected, and housekeepers are at highest risk of OBEs. Moreover, workload intensity and proficiency influence OBE incidence. Every effort should be made to prevent occupational blood exposures through implementation of safe working conditions and education. Although measures to deal with the psychological problems related to OBE are needed, reassurance also seems to be important given the results that show transmission rate is low compared to the frequency of exposure.

## Abbreviations

HBsAg: HBV surface antigen
HBV: Hepatitis B virus
HCV: Hepatitis C virus
HCW: Health care worker
HIV: Human immunodeficiency virus
OBE: Occupational blood exposure
WHO: The World Health Organization

## References

[1] ILO/WHO Joint ILO/WHO guidelines on health services and HIV/AIDS (2005). International Labour Organization and World Health Organization.

[2] Prüss-Ustün A, Rapiti E, Hutin Y (2003). Sharps injuries: global burden of disease from sharps injuries to health-care workers.

[3] Howsepian A (1998). Post-traumatic stress disorder following needle-stick contaminated with suspected HIV-positive blood. Gen Hosp Psychiatry.

[4] Sohn J-W, Kim B-G, Kim S-H, Han C (2006). Mental health of healthcare workers who experience needlestick and sharps injuries. J Occup Health.

[5] Worthington MG, Ross JJ, Bergeron EK (2006). Posttraumatic stress disorder after occupational HIV exposure: two cases and a literature review. Infect Control.

[6] Mannocci A, De Carli G, Di Bari V (2016). How much do needlestick injuries cost? A systematic review of the economic evaluations of needlestick and sharps injuries among healthcare personnel. Infect Control Hosp Epidemiol.

[7] Oh H, Yi S, Choe K (2005). Epidemiological characteristics of occupational blood exposures of healthcare workers in a university hospital in South Korea for 10 years. J Hosp Infect.

[8] Park S, Jeong I, Huh J, Yoon Y, Lee S, Choi C (2008). Needlestick and sharps injuries in a tertiary hospital in the Republic of Korea. Am J Infect Control.

[9] Yun YH, Chung YK, Jeong JS (2011). Epidemiological characteristics and scale for needlestick injury in some university hospital workers. Korean J Occup Environ Med.

[10] EPINet Report for Needlestick and Sharp Object Injuries. at https://internationalsafetycenter.org/exposure-reports/.

[11] Alvarado-Ramy F, Beltrami EM, Short LJ (2003). A comprehensive approach to percutaneous injury prevention during phlebotomy: results of a multicenter study, 1993-1995. Infect Control Hosp Epidemiol.

[12] Benítez RE, Ruiz MA, Córdoba DJ, Escolar PA, López FF (1998). Underreporting of percutaneous exposure accidents in a teaching hospital in Spain. Clin Perform Qual Health Care.

[13] Kotelchuck D, Murphy D, Younai F (2004). Impact of underreporting on the management of occupational bloodborne exposures in a dental teaching environment. J Dent Educ.

[14] Osborn EH, Papadakis MA, Gerberding JL (1999). Occupational exposures to body fluids among medical students: a seven-year longitudinal study. Ann Intern Med.

[15] Makary MA, Al-Attar A, Holzmueller CG (2007). Needlestick injuries among surgeons in training. N Engl J Med.

[16] Prakash K, Patel K (2012). Epidemiology of needle-stick injuries in Mangalore. J Evol Med Dent Sci.

[17] Ayas NT, Barger LK, Cade BE (2006). Extended work duration and the risk of self-reported percutaneous injuries in interns. JAMA.

[18] Saini S, Nagarajan S, Sarma R. Knowledge, attitude and practices of bio-medical waste management amongst staff of a tertiary level hospital in India. Indian J Community Med. 2011;336(2):143–45.

[19] Mathur V, Dwivedi S, Hassan M, Misra R (2011). Knowledge, attitude, and practices about biomedical waste management among healthcare personnel: a cross-sectional study. Indian J Community Med.

[20] Megha K, Daksha P (2012). Knowledge and practices about hospital waste disposal and universal safety precautions in class IV employee. J Commun Dis.

[21] Lee J-J, Kok S-H, Cheng S-J, Lin L-D, Lin C-P (2014). Needlestick and sharps injuries among dental healthcare workers at a university hospital. J Formos Med Assoc.

[22] Marnejon T, Gemmel D, Mulhern K (2016). Patterns of needlestick and sharps injuries among training residents. JAMA Intern Med.

[23] Mansour A (1990). Which physicians are at high risk for needlestick injuries?. Am J Infect Control.

[24] Shefer A, Atkinson W, Friedman C (2011). Immunization of health-care personnel: recommendations of the Advisory Committee on Immunization Practices (ACIP). MMWR Recomm Rep.

[25] Cardo DM, Culver DH, Ciesielski CA (1997). A case–control study of HIV seroconversion in health care workers after percutaneous exposure. N Engl J Med.

[26] Deisenhammer S, Radon K, Nowak D, Reichert J (2006). Needlestick injuries during medical training. J Hosp Infect.

[27] Hofmann F, Kralj N, Beie M (2002). Needle stick injuries in health care-frequency, causes und preventive strategies. Gesundheitswesen (Bundesverband der Arzte des Offentlichen Gesundheitsdienstes (Germany)).

[28] Korean Association for the Study of the L (2016). KASL clinical practice guidelines: management of hepatitis C. Clin Mol Hepatol.

[29] Sulkowsky MS, Ray SC, Thomas DL (2002). Occupational transmission of hepatitis C virus—reply. JAMA.

[30] Eskandarani HA, Kehrer M, Christensen PB (2014). No transmission of blood-borne viruses among hospital staff despite frequent blood exposure. Dan Med J.

